# Decay accelerating factor (CD55) protects neuronal cells from chemical hypoxia-induced injury

**DOI:** 10.1186/1742-2094-7-24

**Published:** 2010-04-09

**Authors:** Ying Wang, Yansong Li, Shawn L Dalle Lucca, Milomir Simovic, George C Tsokos, Jurandir J Dalle Lucca

**Affiliations:** 1 Department of Cellular Injury, Walter Reed Army Institute of Research, Silver Spring, MD 20910, USA; 2 Clinical Research Management, Inc., Frederick, MD 21701, USA; 3 Division of Rheumatology, Beth Israel Deaconess Medical Center, Harvard Medical School, Boston, MA 02115, USA

## Abstract

**Background:**

Activated complement system is known to mediate neuroinflammation and neurodegeneration following exposure to hypoxic-ischemic insults. Therefore, inhibition of the complement activation cascade may represent a potential therapeutic strategy for the management of ischemic brain injury. Decay-accelerating factor (DAF, also known as CD55) inhibits complement activation by suppressing the function of C3/C5 convertases, thereby limiting local generation or deposition of C3a/C5a and membrane attack complex (MAC or C5b-9) production. The present study investigates the ability of DAF to protect primary cultured neuronal cells subjected to sodium cyanide (NaCN)-induced hypoxia from degeneration and apoptosis.

**Methods:**

Cultured primary cortical neurons from embryonic Sprague-Dawley rats were assigned one of four groups: control, DAF treatment alone, hypoxic, or hypoxic treated with DAF. Hypoxic cultures were exposed to NaCN for 1 hour, rinsed, followed by 24 hour exposure to 200 ng/ml of recombinant human DAF in normal medium. Human DAF was used in the present study and it has been shown to effectively regulate complement activation in rats. Neuronal cell function, morphology and viability were investigated by measuring plateau depolarization potential, counting the number dendritic spines, and observing TUNEL and MTT assays. Complement C3, C3a, C3a receptor (R) production, C3a-C3aR interaction and MAC formation were assessed along with the generation of activated caspase-9, activated caspase-3, and activated Src.

**Results:**

When compared to controls, hypoxic cells had fewer dendritic spines, reduced plateau depolarization accompanied by increased apoptotic activity and accumulation of MAC, as well as up-regulation of C3, C3a and C3aR, enhancement of C3a-C3aR engagement, and elevated caspase and Src activity. Treatment of hypoxic cells with 200 ng/ml of recombinant human DAF resulted in attenuation of neuronal apoptosis and exerted significant protection against neuronal dendritic spine loss and plateau depolarization reduction. Furthermore, treatment with DAF resulted in decreased accumulation of C3a, MAC, C3a-C3aR interaction, caspase-9, activated caspase-3, and pTyr416-Src (activated Src) tyrosine kinase.

**Conclusion:**

DAF was found to reduce neuronal cell death and apoptosis in NaCN induced hypoxia. This effect is attributed to the ability of DAF to limit complement activation and inhibit the activity of Src and caspases 9 and 3. This study supports the inhibiting of complement as a neuroprotective strategy against CNS ischemia/reperfusion injury.

## Background

Neuroinflammation and degeneration occurs following hypoxic-ischemic insults such as traumatic brain injury (TBI) or chemical exposure to neurotoxic agents [1]. Neuroinflammation and degeneration often share common pathways frequently leading to neuronal cell death [2]. Complement represents an important mediator during the neurodegenerative process by releasing proinflammatory mediators and anaphylatoxins such as C3a and C5a as well as producing MAC [3]. Complement fragments and C3aR have been demonstrated in normal and ischemic brain tissue [4]. Complement depletion has been shown to reduce post-ischemic brain injury in rats and mice [4]. It has been suggested that complement activation levels in the central nervous system (CNS) following brain injury might increase after blood brain barrier (BBB) break-down [5,6] and might come from cellular sources such as astrocytes, microglia, oligodendrocytes and neurons in response to cerebral ischemia or brain trauma [1,7,8]. In addition, astrocytes and microglia express complement inhibitors on their membranes to control complement activation and mitigate complement-mediated injury [9]. Neurons express low levels of complement regulators compared to astrocytes and it has been suggested that human fetal neurons have the capacity to spontaneously activate the complement system [10].

Inhibition of complement activation using biologics such as soluble complement receptor type 1 (sCR1), C1 inhibitor (C1-INH), C3 convertase inhibitor (Crry), C5a monoclonal antibodies, and C5a receptor antagonists have been shown to reduce post TBI [4,11]. Complement system can be activated via the classical pathway, such as by IgG activation, or by the alternative pathway, such as by factor B activation [12]. In a recent study, intravenous immunoglobulin (IVIG) was demonstrated to protect the brain against injury from experimental stroke in mice [4]. Therefore, targeting the complement cascade represents a potential treatment strategy for the management of ischemic brain injury.

Decay-accelerating factor (DAF, also known as CD55), a ubiquitously expressed intrinsic complement regulatory protein, inhibits complement activation by inhibiting the function of C3/C5 convertases in both the classic and alternative pathways thereby limiting local C3a/C5a and MAC production [13]. Human NT2-N neurons constitutively express DAF which is down-regulated after severe hypoxia and subsequent reoxygenation with human serum [14]. It has been previously shown that increased expression of DAF plays an important role in the reduction of cerebral damage by steroids after Traumatic Brain Injury [15]. It has been demonstrated that inhibition of complement activation by human recombinant DAF results in local and remote tissue protection during mesenteric ischemia/reperfusion in mice [16]. A common model of chemical ischemia in cultured cells involves exposure to cyanide [17]. In the present study, we evaluated the effect of recombinant human DAF on cultured embryonic rat primary neurons subjected to chemically induced hypoxia. Harris et al. 2000 reported that neither human nor rodent DAF are species restricted meaning they can regulate both homologous and heterologous complement activation, suggesting cross-reactivity between human recombinant DAF in rodent preparations [18]. Results indicate that 200 ng/ml of DAF treatment protects rat neurons from injury by suppressing the complement cascade as well as by inhibiting the activation of caspase and Src tyrosine kinase.

## Methods

### Primary neuron culture and experimental groups

An established chemical hypoxia model was chosen that mimics ischemia via chemical manipulation with NaCN (17, 19). Sprague-Dawley rat cortex was dissected at embryonic day 17, dissociated in Ca^2+ ^and Mg^2+^-free Hanks balanced salt solution containing 0.125% trypsin for 20 min. Cells were plated and cultured in 10% FBS/DMEM with humidified 5% CO_2 _incubator at 37°C overnight then replaced the medium with serum-free neurobasal medium containing B27 supplement (Invitrogen, MD). Those cells were treated with 3 μM cytosine arabinoside (Sigma) after DIV 3 for 24 h and replaced with Neurobasal supplemented with B27 and maintained for 12 ~ 18 days. Approximately 90% of the cultured cells were neurons, verified by neuronal marker neurofilament 200 (NF-200), the remaining cells labeled positive for GFAP, indicating that they were astrocytes. Cultured primary cortical neurons (12-18 days) were assigned to of four groups: 1) Control: cells incubated with normal basal medium; 2) DAF Treatment Alone: cells treated with normal basal medium in the presence of 200 ng/ml of recombinant human DAF for 24 hours; 3) Hypoxia (NaCN): cells exposed to 1.5 mM of NaCN for 1 hour in glucose-free neurobasal medium [19], rinsed, then followed by normal basal medium for 24 hours; 4) chemical Hypoxia (NaCN) +DAF Treatment: cells exposed to 1.5 mM of NaCN for 1 hour in glucose-free neurobasal medium, rinsed, then followed by normal basal medium with 200 ng/ml of recombinant human DAF for 24 hours.

### Complement expression and distribution

#### Antibodies and reagents

Recombinant human DAF was obtained from R&D systems (Minneapolis, MN). NaCN, CNQX, D-AP5, and mouse anti-NF-200 antibody were from Sigma-Aldrich, Inc. (St. Louis, MO). Chicken anti-mouse C3a and goat anti-chicken IgY (H&L) antibodies were from Abcam Inc. (Cambridge, MA). Mouse anti-rat MAC primary monoclonal antibody was provided as a gift by Dr J. Pippin (University of Washington). Mouse anti-human C3, and goat anti-mouse DAF antibodies were purchased from Santa Cruz Biotechnology Inc. (Santa Cruz, CA). Mouse anti-rat C3aR antibody was from Cell Sciences (Canton, MA). Anti-Caspase 9, Anti-cleaved Caspase 3 (activated Caspase 3) and anti-Tyr416-Src antibodies (activated Src) were purchased from Cell Signaling (Danvers, MA). Goat anti-mouse Alexa Fluor 488-, goat anti-rabbit 594-conjugated secondary antibodies, and ProLong Gold antifade reagent were from Invitrogen (Carlsbad, CA).

#### Western blotting

Cells (2 × 10^6^) were lysed in RIPA buffer. The Cell lysates were separated on SDS gel according to the manufacturer's protocols (Invitrogen Corp.). The proteins were then transferred onto PVDF and blotted with various antibodies as indicated in the figures. Protein bands were detected by chemiluminescence reagents (Amersham Biosciences).

#### Immunofluorescent staining

Cells were seeded on a poly-D-lysine coated cover slip of 12 mm diameter at a density of 1 × 10^5 ^in 24-well plate. After chemical hypoxia, cells were fixed with 4% paraformaldehyde for 30 min, permeabilized with 0.1% Triton X-100 for 10 min, and blocked with 2%BSA in PBS for 30 min. Cells were stained with primary antibodies for 1 hour. After washing, cells were incubated with corresponding Alexa Fluor 488/594-conjugated secondary antibodies for 1 hour. Stained cells were mounted with SlowFade gold antifade reagent with DAPI, sealed with nail polish, and observed with a confocal laser scanning microscope (Radiance 2100, Bio-Rad Laboratories, Hercules, NJ). Negative controls were obtained by substituting the primary antibodies with corresponding immunoglobulin isotypes.

### Evaluation of cell viability

#### MTT reduction assay

Cell viability was determined by 3-(4, 5-Dimethyl-2-thiazolyl)-2, 5-diphenyl tetrazolium bromide (MTT) assay (ATCC, Manassas, VA). Cortical cells were seeded with density of 2 × 10^4 ^in a 96-well plate then cultured for 12 days. 10 μL of MTT stock solution (10 mg/ml) was added to the hypoxic cells and incubated 2 hrs at 37°C. The resulting MTT formazan was extracted with 100 μL of detergent. The reaction product was analyzed at 570 nm with a microplate spectrophotometer (Spectra max plus, Molecular Devices, CA).

#### TUNEL assay

Apoptotic cells were determined by terminal deoxynucleotidyl transferase dUTP nick-end labeling (TUNEL) assay. TUNEL staining was performed according to the manufacturer's protocol. Cells were fixed with 4% paraformaldehyde. Apoptotic cells were identified by streptavidin-fluorescein detection of biotinylated dUTP incorporation (TUNEL assay, R & D Systems, Minneapolis, MN). Stained slides were visualized by confocal laser scanning microscopy. Positive cells were counted and considered as apoptotic cells.

### Evaluation of cellular function

#### Electrophysiological recordings

Whole-cell recordings were made from 14-18 days cultured cortical neurons at room temperature. Cover slips containing cells were transferred to a small stage mounted on an inverted microscope (Diaphot, Nikon) and were superfused with extracellular saline solution (ACF) containing (in mM): 137 Na-Isethionic acid, 4 K-gluconate, 1.8 CaCl_2_, 1 MgCl_2_, 10 HEPES, 10 glucose (pH 7.4 with NaOH). Recording electrode pipettes had resistances of 2-4 MΩ and were filled with an internal pipette solution containing (in mM): 130 K-gluconate, 1 MgCl_2_, 5 EGTA, 5 MgATP, 10 HEPES and 0.4 Na_2_GTP (pH 7.2 with KOH). Action potentials were evoked by injecting depolarization current into primary cortical neurons under normal conditions and recovery after treatment with 1.5 mM NaCN for 30 mins.

Recordings were not performed during NaCN treatment because the Ag-AgCl reference electrode can be oxidized by NaCN which causes a pseudo neuronal membrane depolarization artifact. Data were collected via a patch clamp amplifier (Axopatch 200B), stored on a PC, and analyzed by pClamp 9.0 software (Molecular Devices, Sunnyvale, California). After whole-cell configuration was achieved, series resistance was compensated by 80~90% and monitored periodically. Most cultured cortical neurons had series resistance around 7-8 MΩ (range, 4-13 MΩ). A small percentage of cortical neurons were considered as unhealthy and discarded due to resting membrane potentials less than -55 mV or gradual changes in membrane potential, input resistance, or action potential amplitudes. For current-clamp recordings, a depolarizing current step was injected to induce multiple action potentials. To quantitatively measure the changes in cultured neuronal network activities, the duration of plateau depolarization was monitored in batches of three minute recordings.

#### Dendrite spine count

Cortical cells were seeded on a poly-D-lysine coated cover slip in 25 mm diameter at a density of 3 × 10^5 ^in 6-well plate. Cultured cortical cells were transfected with 2 μg of green fluorescent protein (GFP) plasmid mixed with 0.5 M CaCl_2 _and HEBS solution for 40 min, washed with DMEM, then normal neurobasal medium was added. Two weeks following GFP transfection, cultured cells were placed on a microscope stage incubation chamber with 37°C and 5% CO_2 _control (Wellcome Trust Center for Human Genetics, UK) then filled with ACF buffer 2 hours before image capture. Images were acquired by using an inverted Zeiss LSM 510 META confocal microscope with a 40× oil immersion objective and a digital zoom of 3×. Image stack was generated by reconstructing 8 sections at an interval 0.4 μm from each slide. The measurements of spine density were determined by counting spines from the length of a 20 μm secondary dendrite from each individual neuron. The rate of N/N_0 _was used in the statistics. N corresponds to the total number of dendritic spines at each time point and N_0 _indicates the number before NaCN administration.

### Statistics

Statistic analysis was performed using commercially available software (GraphPad InStat). Differences among the groups were determined by one-way ANOVA followed by Newman-Keuls test. Data are expressed as mean ± S.E.M. and p value < 0.05 was considered significant.

## Results

### DAF reverses the reduction of plateau depolarization inhypoxic neurons

**Figure 1:**
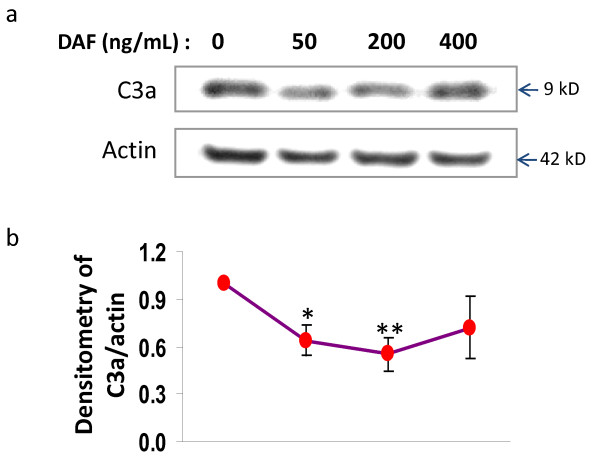
**Recombinant human DAF dose response to C3a generation in cultured primary neurons**. Primary rat cortical neurons were exposed to glucose free medium containing 1.5 mM NaCN for 1 hr, then recovered in normal medium for 4 hrs with 0, 50, 200, 400 (ng/mL) of recombinant human DAF respectively. (a) Cell lysate was immunoblotted with anti c3a and anti β-actin antibodies (n = 5). (b) Data were quantified by densitometry. (one-way ANOVA followed by Newman-Keuls test. * p value < 0.05, **p < 0.01).

To determine the dosage, immunoblotting and confocal microscopic analysis were used to examine the generation of C3a in hypoxic rat primary cortical neurons. DAF displayed a biphasic effect on C3a generation triggered during the exposure of cells to the hypoxic insult (Fig. 1). Within the 50 to 200 ng/ml range, recombinant human DAF was able to suppress C3a production in a dose-dependent manner. Significant inhibition of C3a was apparent in the presence of 50 ng/ml of DAF and reached a maximal level at 200 ng/ml. Interestingly, higher doses of DAF (400 ng/ml) did not show complement inhibition. Accordingly, 200 ng/ml of DAF was chosen to evaluate the function of DAF in suppressing complement activation and neuroprotection.

**Figure 2:**
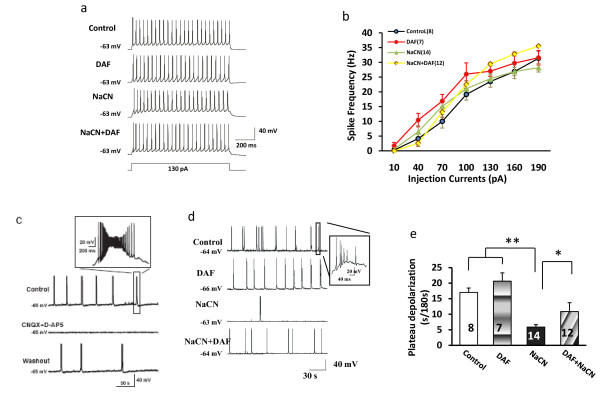
**Effect of DAF and NaCN on neuronal excitability and plateau depolarization in cortical neurons**. Rat primary cortical neurons were cultured for 14-18 days before electrophysiological recording. (a) Effect of DAF and NaCN on action potential discharge in cortical neurons. Repetitive firing was evoked by long depolarization pulse (130 pA, 1 second). (b) Spike frequencies induced by injecting different depolarization currents. Spike frequency is defined as the number of spikes per sec. (c) Neural network activity reflected by plateau depolarization. The representative traces show AMPA- and NMDA-mediated spontaneous plateau potential and burst firing. Insert shows spontaneous plateau potential and burst firing at a much higher time resolution. (d) Effects of DAF and NaCN on plateau depolarization. The typical recordings showing NaCN induced a remarkable reduction in spontaneous plateau potential and burst firing. (e) The spontaneous plateau depolarization was monitored over a three minute period. The data is presented as mean ± SEM. The number of cells recorded is indicated in parentheses and columns (b and e). (one-way ANOVA followed by Newman-Keuls test. *p < 0.05 and **p < 0.01).

To establish whether DAF displays beneficial effects on neuronal excitability and activity under NaCN induced hypoxia, whole-cell patch-clamp recordings from rat primary cortical neurons were performed. Action potentials were elicited in whole cell current-clamp recordings. Neuronal action potential discharges were observed in all four groups. No significant difference in repetitive firing evoked by long depolarization pulse (130 pA, 1 second) (Fig. 2a) and spike frequencies induced by injecting different depolarization currents (Fig. 2b) was observed among the groups. Spontaneous plateau depolarization potentials were recorded after 14 days in culture. The plateau potentials with burst firing were inhibited by excitatory glutamatergic blockers of AMPA and NMDA receptors, CNQX (50 μM) and D-AP5 (50 μM) (Fig. 2c). Spontaneous plateau potential with burst firing was used as an index to reflect neural network activity. Spontaneous plateau depolarization potentials were significantly reduced in hypoxic cells (Fig. 2d). However, treatment with DAF profoundly reversed the reduction in plateau depolarization potentials induced by NaCN (Fig. 2d). Figure 2e shows that the duration of plateau potential with burst firing was considerably shorter in hypoxic neurons compared to controls whereas DAF appears to have corrected the neural change induced by NaCN. This observation suggests that DAF protects neuronal network activity from adverse effects generated by chemical ischemia.

### DAF prevents dendritic spine loss induced by hypoxia

**Figure 3:**
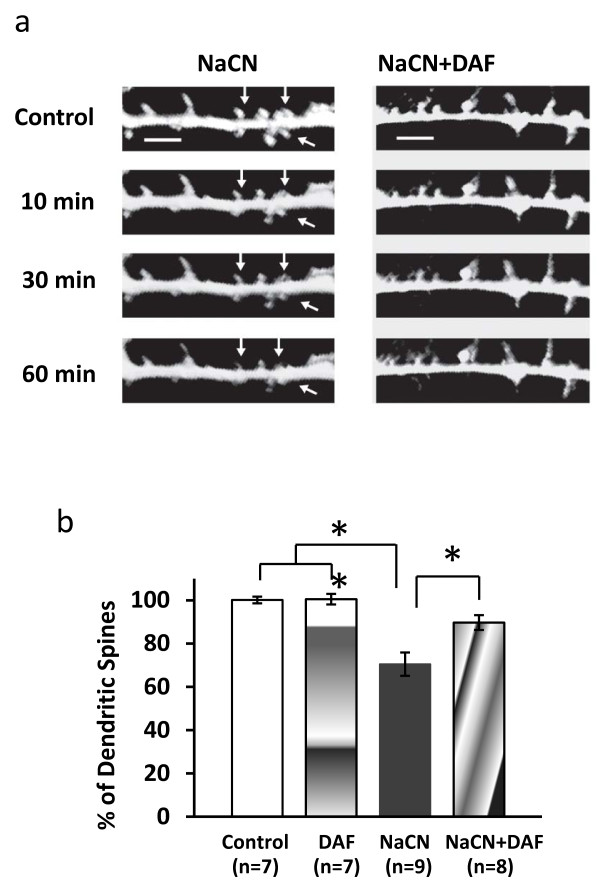
**Time-lapse imaging reveals DAF reduces NaCN-induced dendritic spine loss**. Primary cortical neurons were in ACF buffer with 5% CO_2 _at 37°C for 2 hours before experiment. (a) GFP positive neuron images represent several time points during exposure to 1.5 mM NaCN with and without DAF. Arrow shows spine loss. (b) Images were analyzed by counting dendritic spines prior to and 1 hour after NaCN treatment. Scale bar = 2 μm. (one-way ANOVA followed by Newman-Keuls test. *p < 0.05, **p < 0.01).

Dendritic spine structures and dynamics are important predictors of the function of neural networks [20]. To investigate the potential effect of DAF on morphological changes of neuronal dendritic spines caused by ischemia-like conditions, GFP-transfected neurons were subjected to NaCN and subsequently imaged. The number of dendritic spines in each group was counted. Spine density measurements were determined by counting spines in the length of a 20 μm secondary dendrite from each individual neuron. The rate of N/N_0 _was used, N corresponding to the total number of dendritic spines at each time point and N_0 _indicating the number before NaCN administration. Figure 3a shows time lapse recordings which reveal that NaCN induced morphological alterations which became more pronounced over time. Figures 3a and 3b show that treatment with DAF resulted in significant protection against neuronal dendritic spine loss.

### DAF increases viability and decreases apoptosis of cultured hypoxic neurons

**Figure 4:**
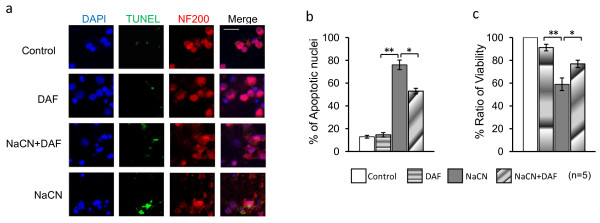
**DAF reduces neuronal cell death induced by ischemia**. Primary rat cortical neurons were exposed to glucose free medium containing 1.5 mM NaCN for 1 hr, then recovered in normal medium with and without DAF for 24 hours. (a) The effects of DAF on the apoptosis of primary cultured neuronal cells as measured by TUNEL assay. DAPI localizes the nucleus (blue), TUNEL shows DNA fragmentation (green), and NF200 shows neurons (red). Positive cells were counted and considered as apoptotic cells (n = 5). Scale bar = 20 μm. (b) The apoptotic cells were counted as described in the methods and the cumulative data from five independent experiments was shown here. (c) The neuronal viability determined by MTT assay (n = 5). (one-way ANOVA followed by Newman-Keuls test. * *p *< 0.05 and **p < 0.01).

To study whether DAF protects neurons from damage induced by ischemic conditions, primary cortical neuron cultures were exposed to glucose-free medium that contained 1.5 mM NaCN. Cyanide selectively produces either apoptosis or necrosis depending on cell type and cyanide concentration [17,21]. Cell viability and apoptosis were assessed by MTT cell proliferation and TUNEL apoptosis assays respectively. MTT demonstrated that exposure of neuronal cultures to NaCN led to 41% more damaged neurons when compared to controls (the control set as 100%) (Fig. 4c). DAF added to the culture medium one hour after the induction of chemical hypoxic ischemia significantly increased cell survival by 19% (Fig. 4c). TUNEL assay was used to determine whether apoptosis occurred after NaCN induced ischemia. Figure 4a demonstrates that DAF reduced ischemic induced apoptosis (DAPI localizes the nucleus, TUNEL shows DNA fragmentation, and NF200 is a neuronal marker). Figure 4b shows that ischemic conditions increased positively labeled neuronal nuclei by 60% when compared to cells from control or DAF groups. This data indicates that this hypoxic-ischemic model mainly triggers neuronal apoptosis, not necrosis. However, the presence of DAF post NaCN insult resulted in a decrease (23%) in the number of TUNEL labeled nuclei. Therefore the beneficial effects associated with DAF on cell viability in this model may be attributed, at least in part, to its ability to inhibit neuronal apoptosis.

### DAF suppresses NaCN-induced C3 protein expression

**Figure 5:**
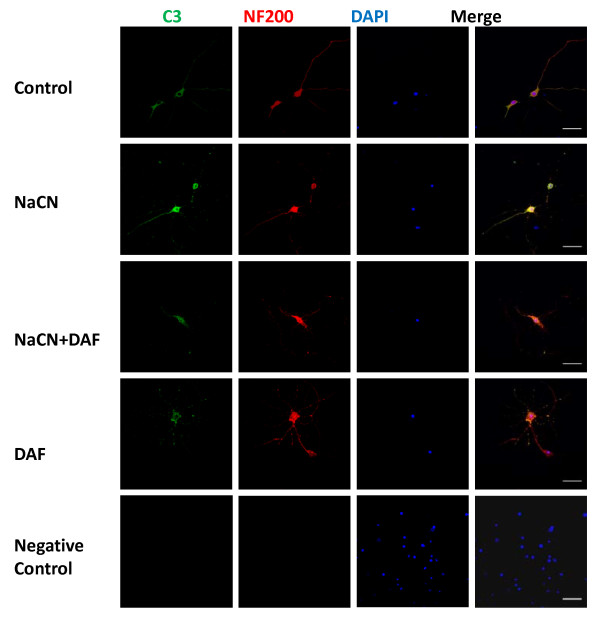
**Treatment with DAF suppresses NaCN induced expression of C3 protein in neurons**. Primary neurons in glucose-free medium were exposed to 1.5 mM of NaCN for 1 hour then the cells were bathed in normal medium with or without 200 ng/ml of DAF. Expression and distribution of C3 in the neurons was assessed by immunofluorescent staining using anti-C3 (green), anti-NF200 (red), and DAPI (blue) antibodies (n = 3). Original magnification: ×400. Scale bar = 50 μm.

To detect whether neurons constitutively produce C3, immunofluorescent staining with anti-C3 antibody and neuronal marker anti-NF-200 was performed. Cultured rat neurons intrinsically express C3 protein which is accumulated primarily at the membrane and cytoplasm of the neuronal body (Fig. 5). C3 is increased after chemical hypoxic exposure, however DAF treatment significantly attenuated this protein expression.

### DAF decreases C3a and C3aR production, C3a-C3aR engagement, and MAC formation under hypoxic-ischemic conditions

**Figure 6:**
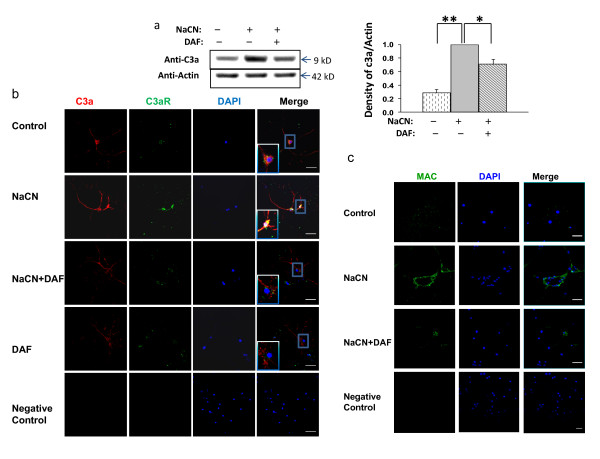
**DAF reduces interaction of C3a-C3aR and MAC formation in rat neurons subjected to hypoxic-ischemic conditions**. Primary rat cortical neurons were exposed to NaCN for 1 hr then allowed to recover for 24 hours in normal medium with or without DAF. (a) Cell lysates were analyzed by immunoblot using anti-C3a and anti-β-actin antibodies. Data was quantified by densitometry (n = 5). (b) Interaction between neuronal C3a and C3aR was determined by immunofluorescent staining using anti-C3a (red) and anti-C3aR (green) antibodies (n = 3). Enlarged images are in the lower left boxes of merged images. (c) Cells were stained with mouse anti-rat MAC antibody (green), DAF resulted in a decrease in MAC formation (n = 3). Original magnification: ×400. Scale bar = 50 μm. (one-way ANOVA followed by Newman-Keuls test. *p value < 0.05, **p < 0.01).

To determine whether DAF interferes with complement activation as it relates to neuronal cells, immunoblotting and confocal microscopic analysis were used to examine the generation of C3a in hypoxic rat primary cortical neurons. Cleavage of the C3 component releases the small peptide anaphylatoxin C3a. Interestingly, soluble C3a (9 kD) was significantly elevated in neurons subjected to hypoxic-ischemic conditions whereas C3a was dramatically inhibited in the presence of DAF (Fig. 6a). To address how soluble C3a associates with neurons, immunofluorescent staining using anti-C3a and anti-C3aR antibodies conjugated with Alexa Fluor 488/594 was conducted. Under hypoxic-ischemic conditions, rat neurons demonstrated a significant increase in C3a generation accompanied by stronger C3aR staining which was observed primarily at the membrane and cytoplasm of the cell body, where colocalization was quite apparent. Treatment with DAF resulted in a reduction of C3a and C3aR expression as well as reduced C3a and C3aR colocalization (Fig. 6b). Cultured neurons exposed to ischemia-like conditions resulted in enhanced MAC accumulation, primarily on the cell membrane (Fig. 6c). In contrast, DAF treatment reduced MAC distribution in response to the insult (Fig. 6c). DAF decreases C3a generation and MAC formation in cultured neurons under hypoxic-ischemic conditions.

### DAF inhibits caspase-3 activation in hypoxic neuronal cells

**Figure 7:**
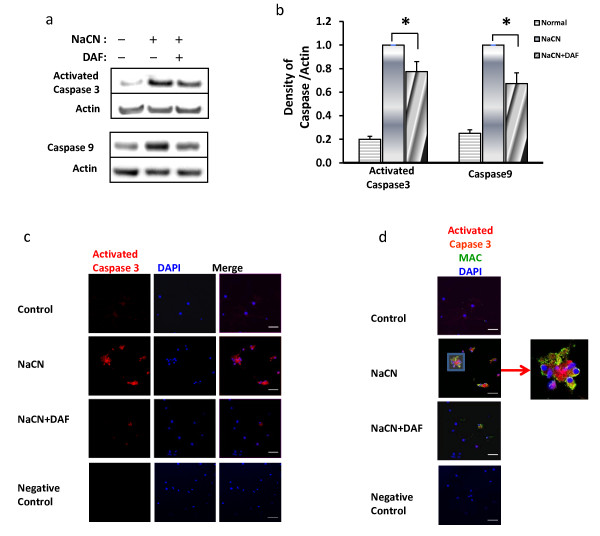
**DAF suppresses the expression of activated caspase in neurons exposed to chemical hypoxia**. Primary rat cortical neurons were exposed to glucose free medium containing 1.5 mM NaCN for 1 hr then allowed to recover overnight in normal medium with or without DAF. (a) Cell lysates were analyzed by immunoblot using anti-active caspase-3, anti-caspase-9, and anti-β-actin antibodies. (b) Data was quantified by densitometry (n = 10). (c and d) Cells were marked with anti-active caspase-3 (red), anti-MAC (green) antibodies, and DAPI (blue) (n = 3). Scale bar = 50 μm. (one-way ANOVA followed by Newman-Keuls test. *p < 0.05).

To examine the effect of DAF on caspase enzymes, activated caspase-3 and caspase-9 expression were monitored by immunoblotting. Hypoxic neurons exhibited strikingly increased expression of active caspase-3 and caspase-9 when compared to neurons cultured in normal medium (Fig. 7a and 7b). However, neurons treated with DAF significantly downregulated hypoxia-induced activation of caspase signaling. This data suggests a novel molecular role for DAF in neuroprotection which involves the suppression of caspases. Additionally, hypoxic neurons displayed strong active caspase-3 staining distributed within the neuronal apoptotic bodies, around fragmented/cleaved nuclei, and at the cytoplasmatic membrane blebbing where they exhibited colocalization with MAC (Fig. 7c and 7d). Conversely, expression of active caspase-3 and colocalization of active caspase-3 and MAC in the plasma membrane blebbing were significantly reduced in cells treated with DAF (Fig. 7c and 7d). These observations imply a potential role of DAF in disrupting the interaction between caspase-3 and MAC in neurons undergoing hypoxia.

### DAF suppresses c-Src activation in hypoxic neurons

**Figure 8:**
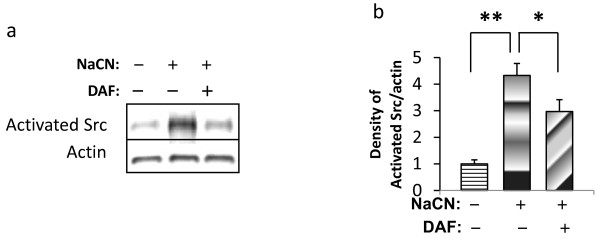
**DAF down-regulates the expression of activated Src in cultured primary neurons exposed to NaCN**. Cells were exposed to glucose free medium containing 1.5 mM for 1 hr then recovered in normal medium for 24 hrs with/without 200 ng/mL of DAF. Protein extractions were immunoblotted with anti-activated Src and anti-β-actin. (b) Data was quantified by densitometry (n = 10). (one-way ANOVA followed by Newman-Keuls test. **p < 0.01, *p < 0.05).

c-Src is extensively expressed in brain cells [22] and is present at much higher levels in neurons than in other brain cells which suggests that it is important to neuronal function. Activated Src plays a pivotal role in neuronal ischemia/reperfusion-mediated injury [23,24]. To further understand the neuroprotective role of DAF in neurons under chemically induced hypoxic conditions, activated c-Src was determined by western blotting using an anti-activated Src antibody. Figure 8 shows that hypoxic neurons displayed higher levels of activated c-Src compared to control neurons. However, DAF suppressed the quantity of activated c-Src induced by the ischemic insult (Fig. 8). These findings imply that DAF-mediated neuroprotection involves inhibition of c-Src activation in neurons exposed to chemical hypoxia.

### Recombinant human DAF can anchor to rat neurons

**Figure 9:**
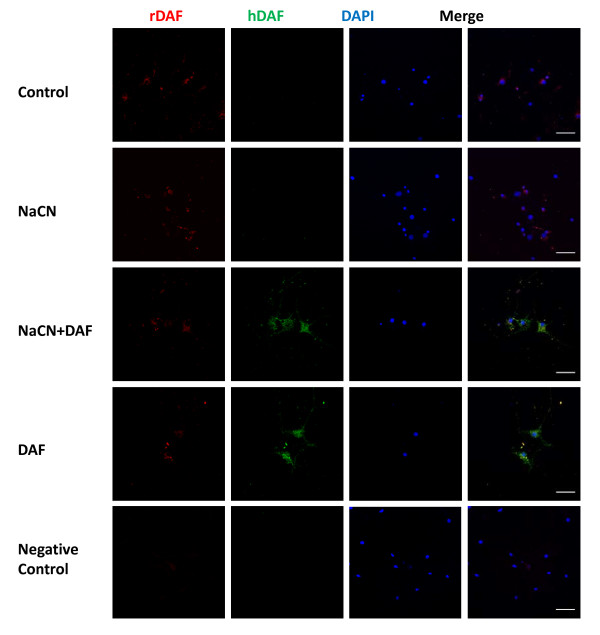
**NaCN does not affect expression of endogenous rat DAF (rDAF) and recombinant human DAF is able to anchor to neuronal cells**. Primary rat neurons were cultured under chemical hypoxic conditions for 1 hour then recovered in normal medium with or without recombinant human DAF. Expression and distribution of rDAF and recombinant human DAF (hDAF) in neurons were shown by immunostaining with anti-rat DAF(red), anti-human DAF(green) antibodies, and DAPI (blue) (n = 2). Original magnification: ×400. Scale bar = 50 μm.

To find out whether recombinant human DAF is able to bind to rat neurons, recombinant human DAF incorporation in cultured rat neurons was determined by immunestaining using anti-human DAF antibody. As shown in Fig. 9, both DAF-treated groups had an obvious recombinant human DAF-stained signal. This staining signal was not observed in control or NaCN groups. Endogenous rat DAF (rDAF) in neurons was also analyzed using immunofluorescent staining. The cultured normal neurons constitutively produced rDAF, but at a very low amount particularly when compared to the level of exogenous anchored recombinant human DAF in DAF treated groups (Fig. 9). This observation is consistent with previous reports [10] suggesting that neurons are susceptible to complement-mediated cellular damage. Although it is noteworthy that complement activation existed (Fig. 6) the overall levels of endogenous rDAF were not significantly altered following chemical hypoxic exposure (Fig. 9). This indicates that a supply of exogenous DAF would be necessary to protect neurons from hypoxic-ischemia insults.

## Discussion

Two major questions have been addressed in this study. First, does the presence of soluble DAF attenuate neuronal damage induced by the chemical hypoxic conditions? Second, how does DAF protect neurons from the hypoxia-mediated injury? The results demonstrate that treatment with DAF protects cellular function and increases cell viability in a cultured neuronal chemical hypoxia model. DAF decreases complement activation and distribution. Furthermore, this protective effect of DAF is associated with the ability to directly inhibit complement activation and suppress (directly or indirectly) Src tyrosine kinase and caspase signaling pathways.

Cyanide is a toxic chemical that has been used as a weapon of war and also as means of terrorist attacks on civilian populations [25]. It inhibits the respiratory chain, blocking the utilization of oxygen and affecting mitochondrial function [17]. Neurons are particularly vulnerable to energy deprivation [26]. Therefore, one of the main target organ system of cyanide toxicity is the central nervous system. NaCN treatment, which mimics acute hypoxic cell damage, is commonly used as an experimental model to study hypoxia in vitro [17]. This model is generally used to measure neuronal viability [27] and provides a means to measure metabolic stress and mitochondrial dysfunction as it relates to excitability of neurons [26]. In addition, NaCN has been used to elucidate mechanisms of ischemic preconditioning [28] and screen for potential neuroprotective compounds/drugs [29]. Here we used NaCN combined with glucose deprivation to mimic a hypoxic ischemic environment in order to investigate the effects of DAF on neuronal injury and to explore the potential mechanisms of DAF associated with neuroprotection.

Electrophysiological changes caused by hypoxic-ischemic conditions are an early sign of neuronal injury and indicator of the degree of injury [30]. Data recorded during cell recovery in normal medium showed that neither NaCN nor DAF changed cortical cell excitability in our model. Spontaneous neuronal electrical activity is critical for many aspects of developmental processes at all stages, such as central axon growth, navigation, and pruning of inappropriate connections [31]. We used glutamatergic AMPA- and NMDA-mediated spontaneous plateau depolarization potentials and burst firing as an index to study neural network activities. The addition of NaCN significantly reduced neuronal spontaneous plateau potential and burst firing whereas DAF reversed these effects. This finding demonstrates that DAF promotes recovery of neuronal network dysfunction induced by hypoxia.

Dendritic spines are micron-sized protrusions of the dendritic membrane that serve as the postsynaptic component for the vast majority of central nervous system excitatory and inhibitory synapses. Spines play a crucial circuit role to synaptic matrix elements of associative neural networks and changes in dendritic spine length or shape have been shown to significantly alter the functional properties of neurons [32]. Dendrite spines can be induced rapidly [33] or reduced and structurally changed due to severe ischemia (within 10 min) and can be observed in the peri-infarct cortex after focal stroke in vivo [34]. In this study, shrinkage of dendritic spines post NaCN administration was apparent as early as 10 min and significant loss of spines was evident at 60 min. Treatment with DAF resulted in a significant reduction of dendritic spine loss. These results suggest that DAF plays a role in preserving synaptic morphology during hypoxia.

It has long been believed that severe or prolonged ischemia/hypoxia leads to neuronal cell apoptosis. Consistent with previous findings [35], when our cultured rat cortical primary neurons were exposed to chemical hypoxia, cell apoptosis was significantly increased. Indirect evidence for the participation of DAF in apoptotic events has been demonstrated in malignant tumors [36] and neutrophils [37]. In this study, DAF-treated cells showed an attenuated level of neuronal cell apoptosis induced by the hypoxic-ischemic insult.

We observed that cultured rat neurons constitutively possessed C3 protein which is consistent with recent findings demonstrating that C3 was present in mouse neurons [4].

In previous studies from our laboratory, DAF was shown to inhibit formation of MAC in murine mesenteric I/R models [[16] and unpublished data] as well as suppress production of C3a and MAC in rodent and porcine hemorrhagic shock [unpublished data]. Of particular interest is our observation that treatment with DAF significantly reduced the increase in C3 expression as well as C3a and MAC formation in hypoxic neuronal cells.

In addition to the well-described regulatory function of DAF on complement activation [13], it has become increasingly apparent that this molecule might also act as a signal-transducing molecule. DAF has been found to associate with Src protein tyrosine kinases such as p56lck and p59fyn in human T cells [38]. There is strong evidence demonstrating that cerebral ischemia/reperfusion induces Src activation in rat hippocampus [39]. Furthermore, blockade or deficiency of Src activity minimized brain injury following stroke in mice [23]. Src family kinase inhibitors PP1 and PP2 decreased brain injury induced by intracerebral hemorrhage, ischemia/reperfusion and neurosurgical procedure [23,40]. In the present study, we demonstrate that DAF treatment significantly decreases the activation of c-Src during ischemia-like conditions. Although the mechanism by which DAF regulates activation of Src kinase is unknown, there is increasing evidence that Src family kinases act as a point of convergence for various signaling pathway, including the pathway that signals via G-coupled receptors [41]. Thus, it is possible that the inhibition of c-Src activity by DAF could be via a direct association of DAF and c-Src.

Neurons have been identified as the principal CNS cell that prominently expresses the C3a receptor under physiological conditions [4]. Significant up-regulation of C3aR in murine brain after cerebral ischemia has been observed [42]. We found that DAF dramatically diminished neuronal C3aR induced by the hypoxic-ischemic conditions. C3aR specifically binds with high-affinity to C3a and high-affinity binding sites are abundantly expressed (20,000 to 80,000 sites per cell) on cultured human astrocytes [43]. The finding that increased C3aR was present in hypoxic cultured neurons and was associated with C3a is quite important since this can explain why soluble C3a was detected in the cell lysates. C3a-C3aR interaction was markedly reduced in the presence of DAF by limiting expression of C3aR and C3a. C3aR is a G protein-coupled receptor which initiates intracellular signaling when C3a binds to it [44,45]. Our findings suggest that autocrine/intracrine C3a might be involved in the regulation of neuronal functions via binding to C3aR and subsequent C3a-C3aR engagement which implies a key link to the down-stream Src and caspase signals.

Sublytic MAC may initiate cellular signal transduction pathways resulting in activation of cell survival mechanisms [46]. However, clinical and experimental studies have implied a pivotal role for MAC in the pathogenesis of secondary neuronal cell death after TBI [47,48]. In our study, an increase in MAC was associated with neuronal injury suggesting lytic formation of this complex due to hypoxia is associated with cytotoxicity and subsequent cell death in this model. Taken together these observations suggest that the protective effects of DAF are related to attenuation of C3a-C3aR-Src/caspase and/or MAC-Src signaling pathways. However, this assumption is speculative and needs further investigation.

The central components of the apoptotic processes are the caspases. Cross-linking of DAF isoform with its antibody in human stomach adenocarcinoma cells elevated the expression of caspase-3 and caspase-8, and activated caspase-3 [49]. But in hypoxic cultured neurons, we observed that application of DAF down-regulated the expression of caspase-9 and reduced caspase-3 activity. Lytic levels of MAC can trigger caspase signal pathway resulting in cell lysis or apoptosis [50,51] therefore it is very likely that in this model, DAF functions by downregulating caspase at least in part by blocking MAC formation. Indeed, we found that treatment with DAF diminished the colocalization/interaction of active caspase-3 and MAC caused by hypoxic conditions. This finding suggests that in addition to suppressing complement activation and Src kinase activity, DAF exerts its neuronal protective effect against hypoxia through a direct or indirect blockage of the caspase pathway.

The present study utilized cultured chemically hypoxic primary cortical neurons as a model of neuronal injury. Extrapolation of our findings to support pharmacotherapeutic innovation for the treatment of ischemic brain diseases should be weighed carefully. First, the model does not account for the role of other cellular components known to play a role in cerebral damage after ischemia and/or hypoxia. Astrocytes, oligodendrocytes and microglial cells have been reported to provide major sources of local complement activation during brain injury [4,8]. Second, studies on Src family kinase signaling in models of cerebral ischemia have revealed that ischemia induces an increase in tyrosine phosphorylation of n-methyl-d-aspartate (NMDA) receptors (NMDAR) by Src family kinases [52,53] suggesting that enhancement of Ca^2+ ^entry induced by the phosphorylated NMDARs or other proteins in the NMDAR complex may be important during activation of intracellular signaling cascades leading to cell death. Our study suggests that DAF interferes with complement activation, but it does not exclude the involvement of other DAF functions such as direct regulation of mitochondrial factors (e.g. apoptosis inducing factors), calcium signaling, NMDAR signaling, or actin cytoskeleton. Future studies will be necessary to determine whether DAF exerts a direct effect beyond complement inhibition on c-Src, NMDARs, transcription factors and caspases, and if so, to what extent these direct interactions contribute to the protective effects of DAF against neuronal damage during ischemia-like conditions.

## Conclusion

Our data indicate that neuronal injury induced by chemical hypoxic insult can be prevented by DAF at the level of neuronal network, dendritic spine morphology, and neuronal apoptosis. Moreover, in addition to complement and caspase pathways, our data also suggests that DAF exhibits neuroprotection through down regulation of Src activity.

## Competing interests

The authors declare that they have no competing interests.

## Authors' contributions

YW participated in the experimental design, performed primary cell culture, TUNEL, MTT assay, live cell image and, and Western-blot. YL participated in the experimental design, performed immunofluorescent staining experiments, data analysis, manuscript revision and formatting. SLDL revised and edited the manuscript. MS helped draft the manuscript. GCT provided critically important intellectual revision. JJDL conceived the study, participated in its design and coordination, wrote and gave final approval for manuscript submission. All authors read and approved the final manuscript.

## Disclaimer

Research was approved by the Institutional Animal Care and Use Committee and was conducted in compliance with the Animal Welfare Act as well as other federal statutes and regulations relating to animals and experiments involving animals and adheres to principles stated in the Guide for the Care and Use of Laboratory Animals, NRC Publication, 1996 edition.

The opinions or assertions contained herein are the private views of the authors and are not to be construed as official or reflecting the views of the US Department of the Army or The US Department of Defense.
